# Leveraging Big Data and Analytics to Improve Food, Energy, and Water System Sustainability

**DOI:** 10.3389/fdata.2020.00013

**Published:** 2020-04-28

**Authors:** Joshua Pitts, Sucharita Gopal, Yaxiong Ma, Magaly Koch, Roelof M. Boumans, Les Kaufman

**Affiliations:** ^1^Global Development Policy Center, Boston University, Boston, MA, United States; ^2^Department of Earth & Environment, Boston University, Boston, MA, United States; ^3^Center for Remote Sensing, Boston University, Boston, MA, United States; ^4^Afordable Futures, Charlotte, VT, United States; ^5^Department of Biology, Boston University, Boston, MA, United States

**Keywords:** FEWs, GIS, sustainability, decision support systems models, ecosystems

## Abstract

With the world population projected to grow significantly over the next few decades, and in the presence of additional stress caused by climate change and urbanization, securing the essential resources of food, energy, and water is one of the most pressing challenges that the world faces today. There is an increasing priority placed by the United Nations (UN) and US federal agencies on efforts to ensure the security of these critical resources, understand their interactions, and address common underlying challenges. At the heart of the technological challenge is *data science applied to environmental data*. The aim of this special publication is the focus on big data science for food, energy, and water systems (FEWSs). We describe a research methodology to frame in the FEWS context, including decision tools to aid policy makers and non-governmental organizations (NGOs) to tackle specific UN Sustainable Development Goals (SDGs). Through this exercise, we aim to improve the “supply chain” of FEWS research, from gathering and analyzing data to decision tools supporting policy makers in addressing FEWS issues in specific contexts. We discuss prior research in each of the segments to highlight shortcomings as well as future research directions.

## Introduction

Human sustainability is one of the most pressing issues of the 21st century. Humans alter the ecological, hydrological, and thermal characteristics of their environment through deforestation, urbanization, transportation, modifications to landform and vegetation cover, and many other infrastructure activities, resulting in an inextricable coupling of human and natural systems. Continued population growth, climate change, and development have increased the intensities of natural resource use and environmental degradation to levels that threaten the stability and security of food, energy, and water system (FEWS) services. Unsustainable resource use leads to biodiversity loss and natural resource degradation, with significant impacts on vulnerable populations.

The Food, Energy, Water Nexus is a UN^1^ framework emphasizing the interdependence between food, water, and energy. Pressures or development in one area will affect the others (Biggs et al., 2015; Leck et al., 2015; Smajgl et al., 2016). The study of FEWS's interactivity is increasingly adopted as a framework for research, technology, and policy to address ongoing sustainability concerns that require multidisciplinary understanding of feedback loops and interactions between human and natural systems (Hussey and Pittock, 2012; Ringler et al., 2013). FEWS studies demand new and innovative scientific approaches from complexity science that can address the integrative, complex, and multi-scale interdependencies across space and time and the dynamics of their interactions (Ostrom, 2007, 2009; Bazilian et al., 2011). FEWS research focuses on understanding the interconnections among each system to derive a comprehensive understanding of the causes and consequences of changes within and across aspects of those systems. If the goal is to generate a sustainable system, the FEWS nexus represents points of overlap or conflict among the elements of those systems necessary to create those outputs, with the ultimate orientation toward “increasing efficiency, reducing trade-offs, building synergies and improving governance” across the systems (Hoff, 2011). The nexus approach seeks to identify mutually beneficial options (i.e., “win–wins”) across various stakeholders, including different human-use sectors, management bodies, civic groups, and public–private partnerships.

Zaidi et al. describe two general techniques to modeling and analyzing FEW systems: *process based* and *data driven* (Zaidi et al., 2018). Process-based approaches rely on well-defined mathematical relationships between known variables within a system. Process-based models were historically favored when a relationship was well-known between variables, but there may be a lack of data availability. Hydrological models, for example, frequently rely on physics laws of water movement (Jain and Singh, 2017). By contrast, data-driven approaches do not typically rely on underlying assumptions between variables but do require a substantial amount of data as inputs, which may be of limited availability depending on geography or context. Typically, data-driven approaches are also quicker to develop and less prone to error and uncertainty often found in process-based models (Jain and Singh, 2017).

Only in recent years have data quality and availability increased sufficiently enough to apply rigorous data-driven analytics methods to more accurately understand the FEWS nexus, making this a big data problem. Further, a comprehensive analysis depends on knowledge and integration of several different data types and sources. These include dynamic georeferenced datasets, and ecological, economic, and social processes based on multi-scale, multi-temporal, and multi-source data. Remote sensing has become one of the most reliable and consistent data sources for addressing societal problems in the past decades.

With the use of quantitative approaches, the data can inform models of ecosystem service flows and trade-offs to demonstrate what is lost and what is gained under decision-making scenarios. FEWS scenarios effectively allows for more accurate “what if” analyses. Moreover, modeled trade-offs can be projected over space and time, providing essential information to guide long-term sustainability analysis and planning. For example, *what is the impact of three different dam construction configurations on fish migration and fish biomass in a time span of 5 years*? The trade-off analysis may enable the decision maker to choose the dam configuration with the least reduction in fish biomass traded off against the total number of fish species impacted over a 5-year period. The scenario analysis has to be presented using the right decision tools by way of visualizations. Despite the need for this systems-level understanding, the collection, integration, and synthesis of data pose challenges owing to different resolutions, coverage, accuracy, and standards. Other practical challenges are that FEWS policies have to service various stakeholders, leading to a need for decision support systems requiring expertise in translating science into policy and action.

As a case study in applications, this paper highlights our ongoing work in Southeast (SE) Asia framed in the FEWS context. SE Asia is a densely populated region consisting of 11 countries occupying 3.6% of the total land area of the Earth, with around 9% of the world's population. More than one half of the population is rural, where agriculture and aquaculture are primary occupations for more than one-third of the total population of this region. SE Asia produces 9.5% of the global market value of agricultural and fishery products. It dominates global palm-oil production (>85%), fisheries (19%), and aquaculture (14%) (Hishamunda et al., 2009; FAO, 2018). In the last two decades, SE Asia has experienced massive deforestation and land transformation owing to overexploitation, agricultural expansion, and conversion to aquaculture. FEWS concerns in SE Asia require an understanding and modeling of systems-level interactions at many spatial scales. In this region, water is at the heart of FEWS, critical to both food (agricultural productivity and fisheries) and energy (hydropower generation, biofuel crop productivity, and cooling in thermo-power stations). Both water quality and supply are mainly dependent on forest ecosystem services as well as climate (precipitation).

**Figure 1:**
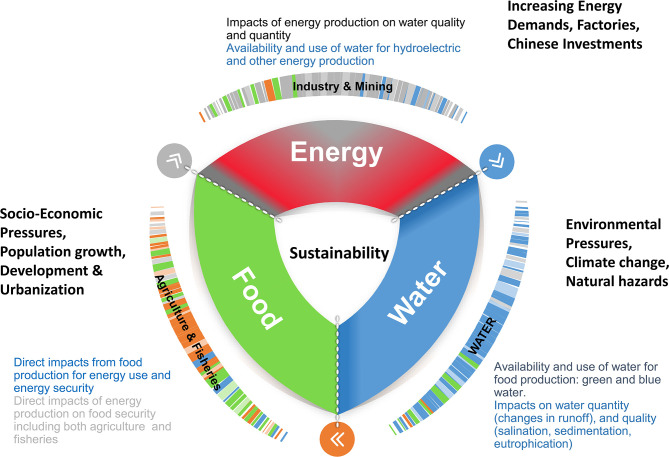
The FEWS (Food, Energy, Water Securities) framework for SE Asia.

Figure 1 shows the FEWS framework for SE Asia. Energy production impacts water quality and quantity, whereas availability of water impacts food production and food production impacts energy production. The connections between the three elements of FEWS require systems approach. The goal is to design and implement a methodology to utilize freely available data, set up systems models, and provide tools for FEWS decision for a variety of stakeholders. We further discuss how the approach can easily be generalized to other regions facing similar FEWS problems.

## Approach and Methods

**Figure 2:**
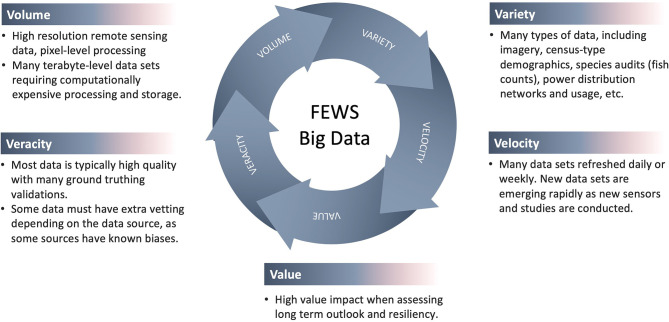
“Big data” is characterized by the 5V's-Velocity, Volume, Value, Variety, and Veracity.

Understanding FEWS nexus depends on knowledge and integration of several different data types and sources. The resulting “big data” is characterized by the 5V's: velocity, volume, value, variety, and veracity (see Figure 2). With the use of quantitative approaches, these data inform models of ecosystem service flows and trade-offs to demonstrate what is lost and what is gained under alternative decision-making FEWS scenarios. Moreover, modeled trade-offs can be projected over space and time, providing essential information to guide Sustainable Development Goals and policy decisions.

With the opening of the Landsat archive in 2008 and the continuation of Landsat and Landsat-like missions (i.e., Landsat 8 and Sentinel-2), we now have access to more observations at moderate resolutions than at any time in the past. As a result, we are now able to explore and more fully utilize the temporal dimension of Landsat data for characterizing surface conditions, allowing us to detect more subtle kinds of environmental change more reliably as well as concurrently as it is occurring. Comprehensive histories of land cover can now be constructed at the full range of spatial extents, ranging from local to global. Specific National Aeronautics and Space Administration (NASA) products related to FEWS include crop models, biophysical models, groundwater, deforestation, carbon sequestration, and climate scenario modeling that provide a wealth of data and analytical products that can aid in transformative and resilient policy and decision making.

**Figure 3:**
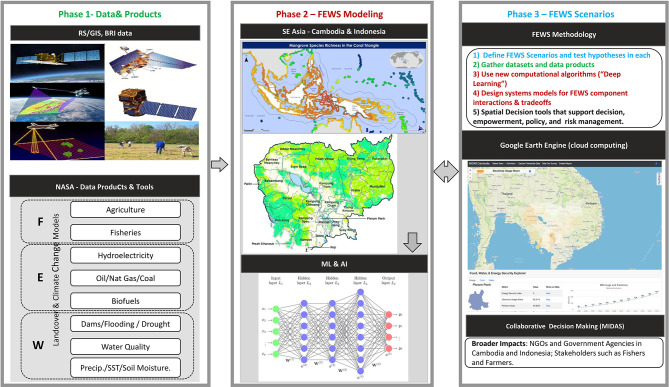
The general framework for FEWS studies in SE Asia.

There are many challenges in using earth science datasets for understanding and monitoring the food–energy–water nexus. These include the following:

1. Challenges that arise owing to the inherent properties associated with this data class, for example, diversity in the type and characteristics of data due to different modes of acquisition.
2. Specialized tools and techniques are needed to process large quantities of data and distill the essential info that can be used in systems modeling.
3. FEWS analysis has to service various stakeholders via user decision support systems requiring expertise in knowing how to translate science into policy and action.
4. FEWS studies need data relating to land cover, hydrology, biodiversity, fish and agricultural food systems, energy production, and economic, social and demographic data in order to build the various scenarios; challenges that arise in developing methods to combine these data types.
5. Expertise is needed to perform the initial data collection, validation, and feature selection to choose the most relevant data to FEWS. The general framework for our approach to FEWS studies in SE Asia is shown in Figure 3, consisting of geospatial data collection and integration, data and analysis, and FEWS policy and decision making.

These challenges and limitations are explored at length in related publications (Eftelioglu et al., 2016).

### Data Types and Inputs

FEWS requires different types of data related to both natural and human systems. Natural system data includes climate, hydrology, soils, land cover, natural hazards, and other terrestrial processes that are captured at a variety of spatial scales. To access these data, there are a number of free data portal tools such as United States Geological Survey (USGS) FEWS NET Data Portal^2^, Thematic Exploitation Platform (TEP) European Space Agency (ESA)^3^, and National Oceanic and Atmospheric Administration (NOAA) View Global Data Explorer^4^ that can guide any user in the selection of relevant imagery from extensive and publicly available data collections (e.g., NASA EarthData). These data portals assist in the selection of satellite time series for the analysis of a range of applications including agriculture, deforestation, fires, land conversion, renewable energy production, and water availability. Geospatial data including GIS, socioeconomic, irrigation, land holding, fishing communities, forestry, and trade) and other data can be obtained from the World Bank and US/European State Department reports and related sites (e.g., Open Development, Cambodia). FEWS studies benefit from utilizing the full spatial, temporal, and spectral domains of Landsat, MODIS, and other sensors and NASA data products to reveal the status and dynamics of human sustainability through the patterns and sequences of land cover change in two countries. FEWS studies can be facilitated through the use of cloud-powered analytical capabilities of Google Earth Engine (GEE) and several space agencies [NASA, NOAA, ESA, Japanese Aerospace Exploration Agency (JAXA), etc.] data products and tools.

The significance of Landsat, MODIS, and ASTER is instrumental in FEWS analysis. Landsat documents the status and dynamics of the Earth's surface, building a continuous time series of space-based reflectance data. Landsat imagery has been used for a multitude of sustainability-relevant applications, including classifying land cover at scales from regional to national and global (e.g., Vogelmann et al., 1998; Townshend et al., 2012), detecting periodic changes in land cover (Hansen and Loveland, 2012), and aiding in understanding social, economic, and ecological processes (e.g., Masek et al., 2000; Cohen and Goward, 2004). In addition to Landsat, FEWS studies can use other publicly available satellite data sources with historical records (from 2000 to present) such as Terra-Aqua MODIS, Terra ASTER, and EO-1 ALI/Hyperion. The JAXA has been engaged since 2009 in producing yearly global datasets of forested and non-forested areas at a spatial resolution of 25 m. These are products generated from JAXA's ALOS-1 PALSAR (2006–20011) and ALOS-2 PALSAR (2014 to present) imagery and are publicly available. Other geospatial datasets include the recently released Shuttle Radar Topographic Mission (SRTM) 1-Arc Second Global Digital Elevation Model (DEM) with improved spatial resolution (30 m) at no cost. Table 1 shows the satellite data sources for this FEWS study.

**Table 1:** Satellite data sources utilized for the study.

	**Sensor(s)**	**Dates of coverage**	**Spatial resolution**
Landsat 1–3	MSS	1972–1983	80 m
Landsat 4 and 5	Landsat TM	1982–2013	30 m (120-m thermal band)
Landsat 7	Landsat ETM+	1999–present	15 m panchromatic, 30 m multispectral, 60 m thermal
Landsat 8 (LDCM)	Operational Land Imager (OLI), Thermal Infrared Sensor (TIRS)	2013–present	15 m panchromatic, 30 m multispectral, 100 m thermal
Terra, Aqua	MODerate Resolution Imaging Spectroradiometer	2000–present	250–5,600 m
Terra	ASTER (VNIR and TIR) SWIR	2000–present 2000–2008	15-m VNIR; 90-m TIR; 30-m SWIR
EO-1	Hyperion, Advanced Land Imager (ALI)	2000–present	10–30 m
Suomi NPP	Visible Infrared Imager Radiometer Suite (VIIRS)	2013–present	375–750 m
Space Shuttle Endeavor	Shuttle Radar Topography Mission (SRTM)	2000	30 m (1 Arc-Second Global)

### Methods

Our FEWS data study for Cambodia includes three products useful for policy and decision making: land cover classification and mapping, coupled systems modeling of hydrological flows over a 30-year period, and estimating FEWS metrics for provinces of Cambodia. Each relies on approaches that are well-suited to problems of big data. Some of these methods are highlighted below.

#### Data Fusion

“Data fusion” represents many different approaches to linking data sources by a common attribute or feature. Because FEWS models are primarily geospatial, the common attribute is often the specific location (e.g., zip code in the USA). Other FEWS data may be only tangentially related to the primary attribute. For example, we include the cost to construct a hydroelectric dam, which we can link to dam locations but does not itself include a spatial component.

Data fusion of satellite images is relevant in land use and land cover classification, environmental monitoring, emergency response, and change analysis. Typically, any single satellite sensor is insufficient to provide all of the benefits offered by combining different sensors (e.g., high spatial but low spectral resolution vs. low spatial but high spectral, and optical vs. SAR). Prior research (Zhang, 2010; Joshi et al., 2016) has explored various methods to optimally fuse these images and evaluate the quality of the image fusion by using clustering to categorize regions in the fused images and comparing the accuracies of the resulting categorization maps. These prior research further discusses how improved results have been achieved by combining principal component analysis (PCA) for multi-resolution images and wavelet transformation to generate multi-resolution representations and information injection. This approach allows for dimensionality reduction to assist with processing tasks and data integration as part of the input used in the Multiscale Model of Ecosystem Services (MIMES) model (*Dam Construction Impacts on Fisheries in the Tonle Sap*) as well as land cover change detection (*Land Cover Change Detection—Urbanization in Siem Reap*). Prior research elaborates on further description and applications of these methods.

We have linked our datasets by common attributes including geospatial coordinates (e.g., lat and long), species, descriptive location (e.g., wetland), and time period. In general, our corpus of fused data is stored in a combination of PostgreSQL and flat files for rapid access across platforms and is able to be handled by Google Cloud Services as well as processed locally as needed.

#### Land Cover Classification

The primary objective of land cover classification is to create spatial maps of the extent of land cover types, for example, forests, water, and croplands, in a given region of study across time. Land cover classification is essential for monitoring the dynamics of diverse ecosystem resources, for example, forests, surface water, and agricultural expanse. However, there are several challenges in developing land cover classification algorithms that can work with heterogeneous remote sensing datasets at multiple spatial and temporal resolutions.

To overcome the challenge of land cover classification using multi-scale data, multi-view learning offers a promising solution because it naturally considers bags of instances as basic units of classification, and every such bag of instances has an associated class label (Dietterich and Flann, 1997). The objective of multi-instance learning is to assign a single label for an entire bag of instances, instead of assigning individual label to every instance, even though features are being observed at the level of instances.

We rely on prior work, which explored variants of multi-instance learning methods for distinguishing informal settlements (slums) from formal urban settlements using very high resolution (VHR) remote sensing datasets (Vatsavai, 2013; Vatsavai et al., 2013). It is essential to use land cover classification approaches that can leverage different views of the data, collected from multiple sensor sources with varying data types and characteristics, for comprehensive monitoring of ecosystem resources. It is worth highlighting that even though ground-truth labels are scarce in some land cover classification problems, there is often an abundance of unlabeled data instances because remote sensing observations are readily available across large geographic regions and over long temporal periods. Several further approaches for multi-view learning have been explored in the existing literature (Sun, 2013; Xu et al., 2013).

#### Change Detection

Change detection (Singh, 1989; Lu et al., 2004; Radke et al., 2005; Canty, 2014) is widely used for rapid assessment of the land use and land cover changes (e.g., deforestation), policy impacts (e.g., energy crops for biofuel development), and changes due to adverse conditions (e.g., forest fires, flood damages to cropland plant, or crop diseases). Most change detection techniques work by comparing observations at the same location (typically a pixel) from pre-event and post-event dates.

Each land cover has unique physical characteristics such as albedo, emissivity, roughness, photosynthetic capacity, and transpiration that significantly influence its spectral reflectance. Information from Landsat's seven spectral bands allows discrimination among many human-dominated and natural land cover types, essentially mapping the status of a study area at any single point in time. Spectral information has been used in its most basic form (i.e., surface reflectance in individual bands), as well as in a variety of band combinations that enhance the discernibility of specific features of interest. An example is the normalized difference vegetation index (NDVI), used to identify vegetated features. Over the last few decades, interest has shifted to exploring the temporal and spatial dimensions of Landsat data. The alteration of spectral properties of the land surface can be identified in Landsat imagery, and many approaches to change detection have been developed (Masek et al., 2000; Hansen and Loveland, 2012).

Random forest classifier is widely used in the classification of remote sensing image (Pal, 2005; Gislason et al., 2006). The method is robust and has been shown to produce good results for ecological mapping and provides better classification results and faster processing (Pal, 2005; Gislason et al., 2006; Corcoran et al., 2013; Du et al., 2015). The random forest algorithm is a combination of decision tree predictors generated based on random vectors sampled independently from the input vector (Breiman, 2001). The algorithm uses a bagging method to generate a training dataset by randomly drawing with *n* replacement, where *n* is the number of features in the original training set. Each decision tree uses binary recursive partitioning to split the input features into heterogeneous groups on the basis of the input variables. The decision tree models are then combined into a “forest” using a voting system. The input features are assigned to the most popular class produced by the forest. This process allows the classification to have significant improvements of accuracy.

#### Google Earth Engine in Food, Energy, and Water System Studies

GEE provides a cloud-based platform for petabyte-scale scientific analysis and visualization of geospatial datasets (Gorelick et al., 2017) and is highly relevant in the FEWS context. A collection of well-documented classification methods was utilized on the GEE platform for imagery analysis. GEE in FEWS studies shifts the burden of data acquisition, data storage, and data processing away from researchers, which allows them to focus on what they do best: research. It may be computationally expensive to ingest external data into GEE. However, many existing algorithms can be implemented in GEE to facilitate more convenient access to computing, storage, and processing. A recent study also supports that GEE can be capable of “big data” processing as well as classification of multi-temporal satellite imagery for crop mapping shortly (Shelestov et al., 2017). The GEE also provides a set of the state-of-the-art classifiers for pixel-based classification for crop mapping, including a neural network.

#### Self-Organizing Maps for Clustering

Self-organizing map (SOM) (Kohonen, 1990) is a popular tool for mapping and clustering high-dimensional data in spatial and ecological sciences (Gopal et al., 2019). SOMs make no assumptions related to distributions of variables or correlations between those variables (Giraudel and Lek, 2001) and hence are suited to FEWS mapping. SOM can incorporate spatial neighborhood and spatial autocorrelation effects that are commonly encountered in GIS and spatial analysis. The clustering method is fast, robust, and visually efficient (in R). We use SOM for denoting the classification of FEWS given an input of food, water, and energy variables.

#### Process-Based Modeling (MIMES)

The Intergovernmental Science-Policy Platform on Biodiversity and Ecosystem Services (IPBES, 2019) is the intergovernmental body that assesses the state of biodiversity and of the ecosystem services provided to society. This agency has listed three ecosystem service models in response to requests from decision makers: Integrated Valuation of Ecosystem Services and Trade-offs (InVEST), ARtificial Intelligence for Ecosystem Services (ARIES), and our model from prior work, MIMES, which is a spatially explicit, dynamic model of ecosystem service flows and trade-offs, used in this paper. MIMES is written in the coding language, Simile (produced by Simulistics Inc.), and has a design and architecture that consists of compartments for “spheres” such as biosphere and atmosphere and compartments for ecosystem services defined within a location (Boumans et al., 2015). Materials, people, services, and locations are modeled through input–output linkages. This architecture provides forecasting in a novel model for the Tonle Sap Lake in Cambodia. MIMES enables us to conduct simulations that are essential to assess various dynamics of interventions or policies in across scenarios (such as “3S dam construction” or climate change).

#### Decision Support Tool (Modeling Integrated Decision Analysis System)

Although not a method in the strictest sense, we have created a web interface tool used by stakeholders around the world to dynamically interact with our analysis and models. This provides for decision support by allowing users to make trade-off decisions and explore different scenarios resulting from our other methods. For example, Modeling Integrated Decision Analysis System (MIDAS) allows for easy construction and modification of simulated water levels, such that additional “what if” scenarios can easily be run through Tonle Sap MIMES. Detailed discussion of the MIDAS tool is outside the scope of this publication.

## Analysis and Results

A FEWS study must be relevant to stakeholders and policy makers to empower decision making. For example, the stakeholders we engage with in SE Asia are interested in (1) FEW decisions, such as siting of dams, forecasting food, or fish production; (2) trade-off analysis to select among alternatives; and (3) estimating FEWS metrics to plan for the future. Discussed below are FEWS products that we developed to aid stakeholders to make specific decisions. First is a scenario trade-off analysis to analyze impacts on fish with proposed dam construction on the Mekong. The trade-off analysis shows that dam construction in the Tonle Sap impacts fisheries and water. Second, we discuss land cover change detection applied to the urbanization effects around world historical sites near Siem Reap, Cambodia. Finally, we discuss FEWS assessment metrics that provides stakeholders a specific estimate of FEWS metrics in Cambodia using available data. The first uses a system, process-based model to aid in scenario and trade-off analysis between fish, water, and energy (hydroelectric). The second product uses machine learning classification methods to study land use changes over time. The last product uses data-driven models and machine learning to classify and quantify FEWS risks for provinces of Cambodia.

### Dam Construction Impacts on Fisheries in the Tonle Sap

One of the largest threats to FEWS outlook in Cambodia in the last decade is the development of hydroelectric dams along the Mekong, in the 3-S river system, and Tonle Sap basin (in Cambodia). These dams threaten the sustained production and delivery of FEWS services, especially to the rural poor who depend upon them most. Furthermore, land cover and land use in Cambodia have changed over the past decades (Davis et al., 2015), impacting its food and water index, economic development, and outlook (Shrestha et al., 2018).

**Figure 4:**
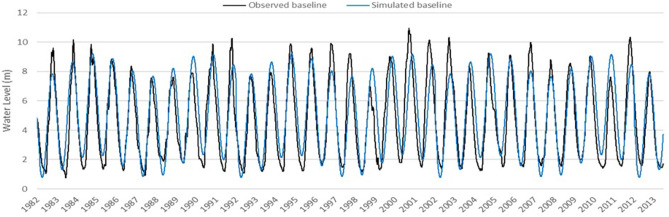
Observed baseline and simulated baseline 1982–2013. Simulated baseline of water levels based on annual and ENSO-like harmonic identified in Fournier analysis.

Figure 3 shows our FEWS approach with input data captured in the left set of boxes. Changes in hydrology are drastically reducing the natural productivity of long-migration fish species, including the commercially crucial (pangasiid) catfishes and that portion of the trey riel catch composed of young-of-year long-distance and medium-distance migratory species. Changes in hydrology are resulting in a major loss of freshwater flooded forest habitat, severely reducing the productivity of resident and short-migration species. Fish monitoring data over the last 7 years is used as input data into our FEWS model with hydrological and climate data. NASA products such as catchment hydrology modeling (VMOD and SWAT-MODFLOW), Landsat Global Inland Water, JRC Global Surface Water Mapping Layers, Global Satellite Mapping of Precipitation, HYCOM, Ocean Color SMI, and PERSIANN-CDR are utilized along with fisheries and other data in a system model to examine trade-offs between hydropower (building dams) and fisheries, as well as climate change and fisheries. We utilize a *process model* called MIMES, as described in *Process-Based Modeling (MIMES)*. Model outputs and analytics include end products for our users such as (1) FEWS risk scenarios, (2) flood pulse in the river during dry and wet season, (3) climate change forecasts into next 10 years, and (4) trade-offs between fishing and dam construction and climate change (see Figure 4).

The hydrology (flood pulse), climate data, land use, population, and fisheries data are input into the model. As this model represents a process-based approach, it utilizes separate data inputs and thus does not require a comprehensive data fusion as described in *Data Fusion*. For example, the *fisheries* “sphere” of the model has definitions, processes, inputs, and outputs specific to *fisheries*. This has relationships to the “sphere” of the model, *population*, which has its own definitions, processes, inputs, and outputs. This is further described in prior work (Boumans et al., 2015).

The total data size for inputs was about 16GB compressed and about 10× this size uncompressed. We ran subsets of the data on subsections of the model in order to optimize and test. Typical desktop hardware was able to run a full simulation in 4–20 h depending on the complexity of the question asked. We utilized cluster resources at Boston University (and later, Amazon Web Services) to run the many simulations in parallel. This was eventually required as we needed to achieve more rapid feedback from our stakeholders and update our outputs.

#### Simulating Lake Pulse in MIMES

MIMES models system dynamics building on prior empirical modeling in the Mekong (Inomata and Fukami, 2008; Arias et al., 2012, 2013). We use habitat groups based on flood regime, physiognomic patterns, and human activity based on prior work (Arias et al., 2012, 2013): open water, gallery forest, seasonally flooded habitats, transitional habitats, agricultural fields, floating rice, and lowland grasslands in 30 hydrological basins. “What if” scenarios in Tonle Sap MIMES are designed to explore the impacts of dams and climate change in this system. Dam impacts include two factors associated with the construction and operation of dams—a reduction in annual flood pulse and the presence of fish migration barriers. These are considered as independent factors within the scenario design. First, dams are expected to reduce the annual flood cycle in this system. For Tonle Sap Lake, this will result in an increase in water levels and the area of permanent flood zone during the dry season and a decrease in the water levels and permanent flood zone during the wet season (on average). To explore this factor, “what if” scenarios that include dam impacts rely on a simulated time series of water levels that reduce the amplitude of the annual flood cycle by 25% (a percentage, which is in the range of the Definite Future dam scenario characterized by the Mekong River Commission, 2015).

Climate change impacts are also evaluated through selected scenarios, such as the expectation that global warming will increase the intensity of the El Niño–Southern Oscillation (ENSO) cycle. The ENSO cycle consists of El Nino and Southern Oscillation impacting precipitation and stream discharge regionally. Prior studies have investigated the connection between ENSO and stream discharge (Enfield et al., 2001; Zhang et al., 2007; Ward et al., 2010). In MIMES, our scenarios evaluate the potential changes in ENSO, which are explored by increasing the amplitude of the ENSO-like cycle associated with the simulated water level input time series. This model and results are further described (Altman et al., 2014; Boumans et al., 2015). One input that drives the model is a time series of water levels, which sets the conditions for the annual flood pulse driving many production processes across the system. In developing and verifying the model, a time series based on observed levels was used, thus allowing for outputs associated with production flows to be compared with available observations of this system. The complexity of outputs can be challenging to understand (Petty, 2014). The model is being validated by stakeholders and researchers in workshops conducted in Cambodia by examining expected outcomes in different scenarios. In contrast, when developing scenarios to explore potential future impacts on hydrology and other factors, a time series of simulated water levels is used. This approach allows us to isolate hydrological factors of critical interest and also to remove sources of unexplained variability in the hydrological cycle. Stakeholders are able to explore these factors though our MIDAS tool discussed in *Decision Support Tool (Modeling Integrated Decision Analysis System)*.

Fourier analysis was used on MIMES outputs to extract harmonic information around time series data—such as amplitude and phase angle—in order to characterize the maximum effect and the time of effect of El Nino on river discharge. Fourier analysis is applied to observed water levels over 30 years on the basis of observed flow in one station on the Tonle Sap. A subset of results is displayed in the Table 2 showing 10 of the top harmonics (52 harmonics were identified in total). We focus on just two harmonics (the ones highlighted) to produce a lake pulse time series for running the model. The first harmonic highlighted represents the dynamics of annual flooding in this system, typically produced by regional monsoons. The second harmonic is a signal that occurs at an interannual scale (about every 5 years) and corresponds roughly to the pattern of greater-than-average flooding followed by drought events that together are often referred to as an ENSO cycle. Using these two harmonics, we produce a simulated time series of water levels—we refer to this as the “simulated baseline” (or just “baseline”), and to which we compare subsequent “what if” scenarios. Figure 4 compares the simulated baseline (produced using only the two harmonics highlighted) to the observed baseline. (The observed baseline was used to develop the model initially including verification and calibration steps using observed data from the system).

**Table 2:** Subset of harmonics.

**Period**	**Frequency**	**Angular frequency**	**Sine**	**Cosine**	**Periodogram**	**Spectral density**
370	0.003	0.017	−2.13	−1.93	33,535	2,565
353	0.003	0.018	1.22	0.9	9,333	2,077
387	0.003	0.016	−0.62	−0.42	2,262	793
339	0.003	0.019	0.43	0.54	1,958	283
406	0.002	0.015	−0.49	−0.44	1,755	131
325	0.003	0.019	0.31	0.39	989	93
2032	0.000	0.003	−0.02	−0.65	1,707	72
428	0.002	0.015	−0.44	−0.1	816	69
313	0.003	0.020	0.41	0.11	721	54
2710	0.000	0.002	−0.09	−0.19	181	44

MIMES also allows for exploration of fish migratory pathways, because dams are expected to have major impacts on the ability of migratory fish species to move freely between spawning and growing areas. MIMES models different fish species on the basis of their migration: periodic long distance and short distance migrators, opportunistic lateral and short distance migrators, and equilibrium migrators. This phase of modeling is being validated by our team.

### Land Cover Change Detection—Urbanization in Siem Reap

Cambodian forest cover declined from 73% in 1993 to between 55% and 60% in 2015^5^. Much of this forest loss is the conversion of forest to urban and agriculture land use. We investigate the rapid urbanization of the area around Siem Reap using Landsat database. Angkor Sites, nominated as United Nations Educational, Scientific, and Cultural Organization (UNESCO) World Heritage in 1992, is located at Siem Reap and draws millions of tourists each year. Prior studies (such as Gaughan et al., 2009) have focused on deforestation from the perspective of tourism and increasing urbanization, whereas others (Evans and Traviglia, 2012) have investigated logging for fuelwood as a major driver for deforestation (Jiao et al., 2015). Decision makers need information on the dynamics of land cover change as well as understand the implications of urbanization. Hence, we utilized a long time series of Landsat data to examine land cover change as well as provide landscape metrics to characterize the nature and magnitude of change. We provide the following products on the basis of relevant metrics: maps and analysis of land cover change, urbanization metrics to characterize the growth, and morphology.

In this study, by using time series Landsat images from 1979 to 2015, we detect and monitor the land use and cover change, and we analyze the spatial–temporal characteristics of the urban morphology and the effects of urbanization on the spatial–temporal distribution of croplands and forest in Siem Reap on the basis of remote sensing and GIS techniques.

We selected six images (1979–2015) from Landsat MSS, Landsat TM, and Landsat OLI (located in WRS II path 127, row 51), ensuring that images have precise radiometric and atmospheric calibration or normalization, similar phonological states, and same spatial and spectral resolution images to facilitate comparison across multi-temporal images. All images were collected during the dry season from December to March to reduce the influence of phonology and were cloud free. The images were ordered from The USGS Earth Resources Observation and Science (EROS) Center Science Processing Architecture (ESPA) and had already been radiometrically calibrated. In order to use directly on classification processes, image registration, layer stacking, and atmosphere correction were applied to the original data using ENVI. The administration map was gathered from Open Development Cambodia (ODC) website. We stacked the satellite images and district boundary data on the basis of the same coordinate system and clipped by district boundary.

We utilized data fusion approaches as discussed in *Data Fusion* to link data by key attributes, including latitude and longitude. Table 1 shows the data sources used for this study. Although we utilize a small fraction of total images for Cambodia from the large corpus of satellite data, we have to process significant data in order to select and crop the areas of interest. For example, for Landsat 4 and 5, we have 7,028 images from December 2, 1987, to November 13, 2013. There are seven bands (roughly 6,900 pixels by 7,700 pixels) in int16 data type. Thus, the entire Landsat 4 and 5 archive utilized is around 5.22TB. For Landsat 8, there is 17.67GB of raw data for every 5 days of coverage. Estimated total data processed each cycle is between 100TB and 200TB. The data storage requirements and processing were achieved on GIS Boston University Cluster resources, and owing to the size of the data involved and the time required to process, it is likely not possible on today's typical consumer desktop hardware. Similar resources, such as Amazon Web Services Elastic storage and Elastic Compute, can be used in place of our cluster resources.

#### Land Cover Classification and Accuracy Assessment

We use multi-instance learning discussed in *Land Cover Classification* with random forests discussed in *Change Detection* to accomplish the classification. We applied the random forest classification method to classify the six images based on randomForest Package from R (Liaw and Wiener, 2002). The training data for our study were collected from corresponding high-resolution Google Earth imagery. All training data were imported into R, and codes were written to implement random forest classification. We identified four land cover classes: *forest, cropland, urban* built-up area, and *other*s. We drew stratified random training samples on the basis of Cochran's stratified random sampling scheme when “simple random sampling is taken in each stratum” (Cochran, 1977, p. 89). Strata in our case represent the four land cover classes. Cochran's equation:

$$\begin{array}{l} n={\left(\frac{\sum w_{i}s_{i}}{S\left(\overset{´}{p}\right)}\right)}^{2} \end{array}$$

where the *w*_*i*_ is the stratum weight, *s*_*i*_ is the standard error for stratum *i*, and *S*(*p*) is the target standard error of the classification. We set the target standard error to 5% to derive a total of 385 random samples (*n*). Time series of the high-resolution satellite images from Google Earth were used as reference data of ground truth; however, owing to the limitation of the date of the images, only classification maps in 2010 and 2015 were assessed for accuracy. The overall accuracy of 2010 is 82.6% and of 2015 is 85.5%, whereas the corresponding kappa coefficient values are 75.2% and 78.9%. We next applied several metrics to describe the urban sprawl dynamics and expansion distribution.

#### Growth Index

The average annual growth index *G*_*ij*_ product describes the average area growth of one type of landscape:

$$\begin{array}{l} G_{ij}=\frac{\Delta U_{ij}}{\Delta T} \end{array}$$

where Δ*U*_*ij*_ is the total area growth in period *i* to *j* and Δ*T* is the time period from *i* to *j* in years. This index gives direct statistical description of landscape area change per year and indirectly reflects the speed of change.

#### Spatio-Temporal Urban Expansion

**Figure 5:**
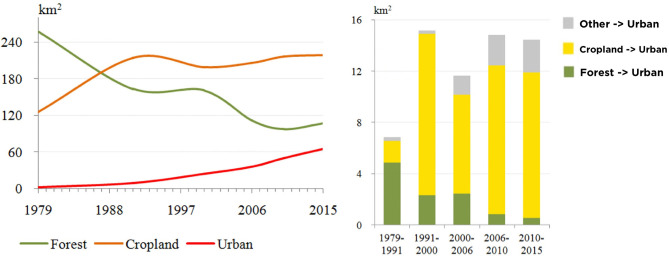
**(Left)** Area change of forest, cropland and urban and built land from 1979 to 2015. **(Right)** Components of new urban area. The y-axis is the total area of urban and built land converted from the forest *(F*→*U)*, cropland *(C*→*U)*, and other *(O*→*U)* between two dates in km^2^.

**Figure 6:**
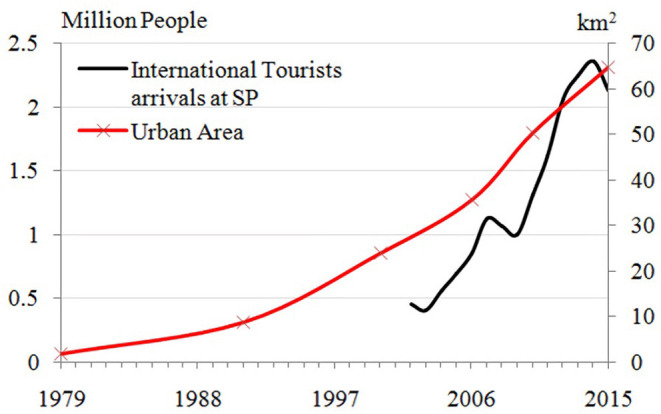
Urban and built land area and tourists population.

**Figure 7:**
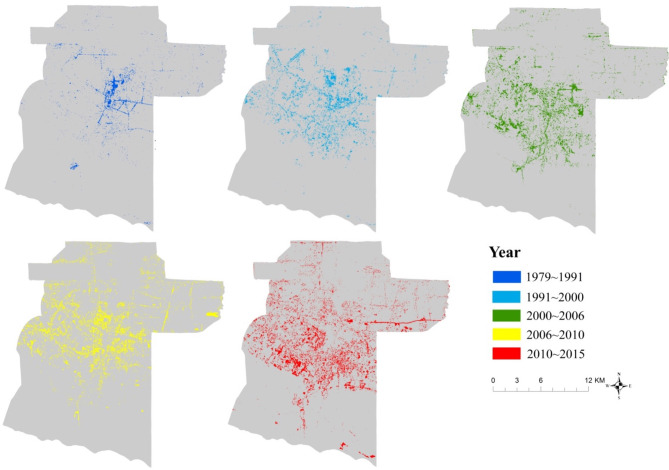
Spatial changes in Urban and Built-up land from 1979 to 2015.

Table 3 and Figure 5 are useful in characterizing urban growth that aids in urban planning and policy. The explosive urban growth in the nineties coincided with the nomination of the Angkor Site as UNESCO World Heritage (in Figure 6), which resulted in the expansion of tourism in the Siem Reap region. Figure 7 shows the urban expansion in various time periods. The spatial pattern of the land cover change into urban seems dispersed but is mainly concentrated in the central and northern Siem Reap region around Angkor Site. Between 1992 and 2000, the expansion of the urban and built land is east–west, following the national highway. More recent changes show a dispersed distribution of urbanization along central Siem Reap. Urban planning can focus on constraining settlement expansion along the highways or around the temples because the seasonal variations of the groundwater table and excessive groundwater depletion are causing the collapse of temple walls (Chen et al., 2017).

**Table 3:** Total urban and built area in km^2^, total urban and built area change in km^2^, and average annual growth index in km^2^ for urban and built.

	**1979**	**1991**	**2000**	**2006**	**2010**	**2015**
Area	1.83	8.67	23.81	35.44	50.27	64.72
Change area	–	6.85	15.13	11.64	14.83	14.45
Average annual growth index	–	0.57	1.68	1.94	3.71	2.89

*The change area and the average annual growth index were calculated in time periods 1979~1991, 1992~2000, 2001~2006, 2007~2010, and 2011~2015*.

We analyze the land cover change from 1979 to 2015 in Figure 7. The overall drop in forest and the corresponding increase in urban and croplands over the time period suggests the increase in urbanization in this area.

Two landscape metrics were also incorporated in the study in addition to temporal analysis: fractal dimension index (*D*) and compactness index (*C*) that describe the urban patch characteristics, useful in urban planning:

$$\begin{array}{lll} D_{ij} & = & 2\frac{\ln\frac{P_{ij}}{4}}{\ln\;A_{ij}}\\ C_{ij} & = & 2\sqrt{\pi \frac{A_{ij}}{P_{ij}}} \end{array}$$

where *P*_*ij*_ is the perimeter of patch *ij* and *A*_*ij*_ is the area of patch *ij*.

Fractal dimension index *D*_*ij*_ reflects the spatial-filling capacity inside the patches and the complexity of the patches morphology by a perimeter-area proportion (McGarigal et al., 2012). The range of *D*_*ij*_ is 1 to 2, and a larger value indicates that the spatial-filling capacity inside the patches is weak, and thus, the morphologic complexity is relatively high. When the value of fractal dimension index is around 1.5, this indicates the form of the patches is on the edge of “Brownian movement,” and the closer the value is, the worse the stability is.

Compactness index *C*_*ij*_ is used to quantitatively measure the clustered nature of urban expansion over time. The city expansion is characterized as being compact or being incompact (Batty, 1991). The range of compactness *C*_*ij*_ is 0 to 1, a larger value represents a higher compactness of the patches, and a smaller value close to 0 suggests the incompact shape of the patch.

The indices in all six time periods is shown in Table 4 and can be interpreted to understand urban expansion and its spatial implications from the policy and planning perspectives. Similarly, the fractal dimension is a low fractal dimension index, suggesting a slow urban creep or sprawl in this region.

**Table 4:** Fractal dimension index and compactness index of urban and built area in Siem Reap through 1979 to 2015.

	**Fractal dimension index**	**Compactness index**
1979	1.47	0.17
1991	1.50	0.13
2000	1.50	0.14
2006	1.48	0.17
2010	1.50	0.18
2015	1.49	0.19

### Food, Energy, and Water System Assessment Metrics

Many agencies provide metrics to describe an integrated summary of variables such as United Nations Development Programme's (UNDP's) Human Development Index (HDI) or the Center for Global Development (CGD) and Foreign Policy's (FP's) Commitment to Development Index (CDI). Prior studies have developed indices on the basis of the study objectives with a focus on water (Daher and Mohtar, 2015; Endo et al., 2015), resource use efficiency (Ringler et al., 2013), sustainable development (Kurian, 2017), and trade-offs (Smajgl et al., 2016). These measures are intuitive and straightforward and enable users to conduct nontechnical comparisons between regions. FEWS is a multidisciplinary issue, and assessments of any measure or metric must incorporate knowledge and insights from multiple disciplines. There is no one index that is likely to be a “catch all,” and a combination of indices that cover security in food, energy, and water concepts is more likely to provide an accurate assessment in the study region. Our FEWS framework is based on available relevant data weighted equally at the province and commune levels (based on data availability). Cambodia consists of 26 provinces and 1,621 communes; each commune can consist of three to 30 villages. The equal weighting of the water, food, and energy sectors is central to our analytical rationale; approaches that bias the weighting of one specific sector, for example, energy resources, tend to constrain analysis to the connections of one or possibly two of the other sectors. We use various input data (described below) and derive FEWS clusters using SOM. Each cluster represents a different nonlinear combination of input variables. Each index is discussed below; each provides a measure of security and outlook, as described.

#### Food Index

The Census of Cambodia provides agricultural data at the province level. The best data are available for the 2011–2013 period^6^. For food index measured at the commune level, we utilize data for rice and maize production in provinces of Cambodia^7^. Rice and maize (corn) are two important crops in Cambodia. Rice (dry and wet season) are included in this estimation of food index. We include fish catch into the food index as well as family consumption patterns, fishing consumption, number of small farmers, livestock, population density, size of the household, and estimated vulnerability to climate change. A higher food index indicates a larger quantity and variety of food, a strong positive outlook over the next decade, and minimal risks to the food supply chain.

#### Water Index

We adopted the water risk (index) from the World Resources Institute product called the Aqueduct Water Risk Atlas^8^. This includes 12 global indicators grouped into three categories of risk and one overall score. Overall water risk identifies areas with higher exposure to water-related risks and is an aggregated measure of all selected indicators from the Physical Quantity, Quality and Regulatory, and Reputational Risk categories. We utilize the overall score for each province of Cambodia derived from World Resources Institute (WRI) global data. Details of the indicators are described in a document as well as the WRI website.

In Cambodia, most communes (located in provinces) have low-to-medium and medium-to-high overall water risks. Koh Kong has the lowest overall water risk value, whereas Svay Rieng has the highest risk, with high risk in baseline water stress, interannual variability, seasonal variability, and flood occurrence. We normalize the values and present the metric.

#### Energy Index

We adopted statistics from International Energy Agency and Electricité du Cambodge (EDC) for the year 2015 that shows the breakup of energy data for Cambodia. Coal accounts for 37%, hydro 38%, imports 22%, and fuel oil (2%), of generation by type in 2015^9^. A World Bank report^10^ notes that 97.6% of Cambodian households have access to at least one source of electricity−71.5% on the grid and 26.1% off the grid, mostly solar home systems and rechargeable batteries.

The country has improved biofuels and has solar energy potential in every province. We note that biofuel and solar potential as well as planned hydroelectricity generation can impact energy index in each province. We estimate the solar potential for communes in each province using estimates solar average irradiation 2007–2015 (kWh/m^2^/day^11^) provided by the World Bank.

We trained our FEWS SOM using 3 × 3 hexagonal grid and then examined the clustering of the communes in the resulting nine clusters. Preprocessing was essential to allow the most informative results for our stakeholders. The finest geographic scale we aimed to achieve was a commune, the scale at which many policy decisions are made in Cambodia. These communes can then be aggregated into other geographic regions such as province. There were approximately 1,600 communes utilized as inputs into our SOM. Various dimensions (number of columns in each vector) were explored, and eventually we included the SOM as part of the postprocessing of MIMES and Land cover changes, as it relied on food-related inputs, which were fish outputs from *Dam Construction Impacts on Fisheries in the Tonle Sap* as well as land cover details from *Land Cover Change Detection—Urbanization in Siem Reap*. This model was run on consumer desktop hardware but would need to be run on a compute cluster if preprocessing is not performed on each vector input.

**Figure 8:**
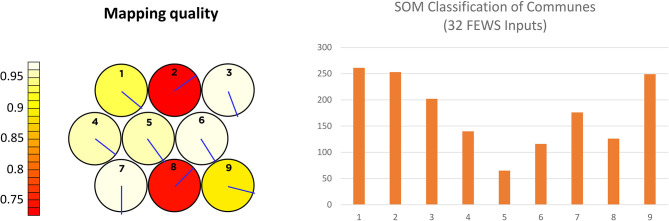
Self-organizing Map (SOM) commune classifications and quality.

We considered all map units individually to produce summaries—quantitative descriptions of data properties. It is worth noting that the goal of SOM is not to find an optimal clustering for the data but to get good insight into the cluster structure of the data for data mining purposes. Figure 8 shows the quality of SOM nodes and the number of communes in each cluster. The best clusters (highest quality in Figure 8) pick up the following trends. One cluster is dominated by rural, agricultural areas with agricultural employment (for both males and females) and no education or infrastructure. Another rural cluster is characterized by rural working men, lack of electricity, and poultry. The third cluster is dominated by secondary and tertiary activities with educated workforce in urban areas such as the capital city, Phnom Penh. The fourth cluster is dominated by primary school educated men and women with small rice farms. Another cluster is dominated by working women in rural areas. SOM clustering shows spatial variation within provinces.

As discussed in *Decision Support Tool (MIDAS)*, we provide a user decision interface called MIDAS to ensure that users visualize and analyze the FEWS challenges spatially, and to support sustainable decision making in the region^12^. Our nexus goal is to ensure that livelihood and well-being opportunities are protected for Cambodians now and into the future. The FEWS tool displays results from various model outputs that enable users to scope and assess a specific problem. MIDAS employs a machine learning segmentation algorithm to classify factors related to FEWS. Each region (province or commune) is segmented into one of nine classes on the basis of the relationships between various FEWS inputs (Figure 8). Users can assess similarities and differences between various regions in terms of FEWS.

Our products described in this section demonstrate the depth and breadth of FEWS analysis required for decision and policy making. It should be emphasized that decision objective making varies across different stakeholders. Although nongovernmental organizations (NGOS) are interested in community livelihoods and health, state and local governments have to consider about sustainability in the future.

## Discussion

Our research methodology provides foundational support in the application of big data science to ecosystem based-management decision making and design, yielding tools necessary to advance FEW sustainability itself. New and faster data algorithms developed will be generally applicable in other systems and will be a useful tool to help local fishers/farmers, NGOS, international development organizations, state, and federal agencies to design and implement sustainable management plans. The FEWS problem is set at the heart of our SE Asia, which can be generalized to other areas and hence work will speak to a wide variety of users. Our research may have far-reaching impacts on how spatial data is accessed and used by global citizenry. For example, non-profits and international groups may use our decision support system for sustaining livelihoods, promoting health and well-being, conserving dynamic biodiversity, and managing sustainable land. State and federal agencies can formulate sustainability plans and make informed decisions on land cover change, biodiversity, deforestation, and sustainable development.

### Future Analysis and Scalability

FEWS approaches will increasingly adopt more “big data” techniques to tackle societal problems in the coming decades. For the MIMES modeling, we anticipate that future research will allow more detailed modeling within the “spheres” of a process model combined with more data available for input. These advances will prohibit standard desktop hardware from being utilized at all—requiring high availability cluster computing to perform the simulations. However, process models are time intensive to develop than is a data-driven model. As more (and more accurate) data are available, we anticipate more reliance on data-driven modeling, which are more scalable, both algorithmically and with human resources. GIS processing and remote sensing approaches discussed in *Land Cover Change Detection—Urbanization in Siem Reap* suffer from fewer issues of algorithmic scaling, as the algorithm is applied to one image at a time. This means that the scaling issues are more around data storage concern, as more images become available at regular intervals. For SOM concerns in the future, a balance must be struck between the size of the input vectors as well as the preprocessing concerns. We elected to preprocess data to produce summary metrics that then fed into the input vectors. However, if different spatial scales are needed, such as if you are looking at parcels in the United States, the number of vectors would be many millions. And if preprocessing was not performed, then input vectors could reach many thousands of columns. It is not clear how well a SOM would perform under these data conditions.

## Data Availability Statement

The datasets generated for this study are available on request to the corresponding author.

## Author Contributions

JP and SG managed the project and wrote the paper. JP contributed to the software design and engineering as well as analysis and writing. YM provided help in analysis and figures. YM and MK sourced NASA and other databases. RB provided MIMES models. LK is the principal investigator (PI) on the project and contributed to the conception of the study, and the entire group helped with constructive discussions and the review.

## Conflict of Interest

RB was employed by company Afordable Futures. The remaining authors declare that the research was conducted in the absence of any commercial or financial relationships that could be construed as a potential conflict of interest.

## Footnotes

^1^http://www.unwater.org/water-facts/water-food-and-energy

^2^https://earlywarning.usgs.gov/fews

^3^https://tep.eo.esa.int/

^4^https://www.nnvl.noaa.gov/view/globaldata.html

^5^Trends, F. (2015). Conversion timber, forest monitoring, and land-use governance in Cambodia. *Forest Trends Report Series*.

^6^Note that Tboung Khmum became a province in 2013 when Kampong Cham Province was split into two in 2013. Hence, some of the 2011 data for this province are derived from Kampong Cham Province.

^7^Source: https://www.nis.gov.kh/nis/CAC2013/Final_Report_En.pdf

^8^https://www.wri.org/sites/default/files/Aqueduct_Global_Maps_2.1.pdf

^9^http://www.edc.com.kh/images/Annual%20Report%202015%20Publish_EnglishVersion.pdf

^10^World Bank (2018). Cambodia beyond connections: Energy access diagnostic report, Washington DC, 15 March 2018. http://documents.worldbank.org/curated/en/141011521693254478/Cambodia-Beyond-connections-energy-access-diagnostic-report-based-on-the-multi-tier-framework

^11^https://globalsolaratlas.info/

^12^http://45.55.215.153/midas/#
